# Effect of vitamin D deficiency on metabolic syndrome among Korean shift workers

**DOI:** 10.5271/sjweh.4072

**Published:** 2023-02-27

**Authors:** Eunchan Mun, Yesung Lee, Woncheol Lee, Soyoung Park

**Affiliations:** 1 Department of Occupational and Environmental Medicine, Kangbuk Samsung Hospital, Sungkyunkwan University, School of Medicine, Seoul, Republic of Korea

**Keywords:** circadian rhythm, Korea, metabolic cardiovascular disease risk, nutritional deficiency, work schedule

## Abstract

**Objective:**

This study aimed to investigate the effect of vitamin D deficiency on metabolic syndrome among shift workers.

**Methods:**

This study included 207 756 workers who underwent a comprehensive health examination at a large South Korean university hospital between 2012 and 2018. We performed multivariate-adjusted logistic regression analysis and analyzed mediation and exposure-mediator interaction.

**Results:**

Overall, 5.5% of the participants had metabolic syndrome. Compared to day work, the odds ratios (OR) of metabolic syndrome and vitamin D deficiency (<12 ng/mL) for shift work were 1.14 [95% confidence interval (CI) 1.06–1.22] and 1.63 (95% CI 1.57–1.70), respectively. Among shift workers, the OR of metabolic syndrome for vitamin D levels of 12–20 and <12 ng/mL, compared with a level of ≥20 ng/mL, were 1.36 (95% CI 1.15–1.61) and 1.51 (95% CI 1.26–1.81), respectively. Shift work and vitamin D deficiency showed an additive interaction; the relative excess risks due to interaction, attributable proportion, and synergy index were 0.26 (95% CI 0.08–0.44), 0.17 (95% CI 0.07–0.28), and 2.09 (95% CI 1.23–3.55), respectively. When vitamin D deficiency was treated as a mediator, the direct and total effects of shift work on metabolic syndrome were 1.12 (95% CI 1.04–1.22) and 1.15 (95% CI 1.07–1.25), respectively. The indirect effect was 1.03 (95% CI 1.02–1.04) and accounted for 18% of the total effect.

**Conclusion:**

Vitamin D is a potential mediator of the impact of shift work on metabolic risk factors.

Metabolic syndrome is defined as a cluster of clinical findings that increase the risk of cardiovascular disease (CVD), such as abdominal obesity, hypertension, diabetes mellitus, and dyslipidemia. Diagnostic criteria have been established to identify individuals at risk of developing CVD. The prevalence of metabolic syndrome has increased over the past three decades, and it was estimated to impact a quarter of the world’s population in 2018 (1).

Several recent meta-analyses revealed that shift work is one of the factors that increases the risk of metabolic syndrome (2–4). Although the underlying mechanism is yet to be fully explored, circadian disruption caused by shift work has been considered as a key factor of metabolic syndrome (5). The circadian system regulates lipid and glucose metabolism throughout the secretion of endocrine hormones, such as insulin, glucagon, growth hormone, and cortisol, as well as the activation of a number of metabolic enzymes and nuclear receptors (6). Blood pressure also exhibits a circadian pattern of nocturnal decrease and diurnal rise, reflecting the circadian rhythmicity of the autonomic nervous activity and renin-angiotensin-aldosterone system (RAAS) (7). Melatonin, an important marker of circadian rhythm, has a preventive effect on metabolic syndrome (8–10), and its secretion might be decreased in night shift workers (11). Nocturnal food intake can induce metabolic disturbance and body mass increase, resulting in metabolic syndrome (12, 13). Another possible factor is short sleep duration that results from shift work, since it can increase the risk of metabolic syndrome components, such as hypertension, diabetes mellitus, and obesity (14).

Recently, the emerging role of vitamin D as an important metabolic regulator in the pathogenesis of metabolic syndrome and its components has been highlighted. Although the relationship between low serum vitamin D levels and obesity is most probably due to volumetric dilution (15). Other proposed mechanisms, such as vitamin D sequestration or altered vitamin D metabolism in adipose tissue and impaired hepatic 25-hydroxylation of cholecalciferol, may also contribute to it (16–19). Regarding lipogenesis, vitamin D and vitamin D receptor interfere in adipocyte maturation by inhibiting the expression of peroxisome proliferator-activated receptor-gamma (PPARγ) and PPARγ-dependent gene expression (20, 21). Vitamin D is also related to blood pressure, since vitamin D is a potent suppressor of renin biosynthesis and can affect regulation of the RAAS (22). In addition, several studies reported that a low level of vitamin D triggers the development of insulin resistance by molecular actions, such as mediating vitamin D–responsive gene transcription or inducing chronic inflammation in adipose tissue, and results in type 2 diabetes mellitus (23–27).

At least 90% of the body’s vitamin D requirement is achieved through endogenous synthesis in the skin via exposure to ultraviolet-B (UVB) (28). Since shift workers are likely to suffer from vitamin D deficiency due to low exposure to sunlight (29), they might be at an increased risk of metabolic syndrome. Nevertheless, there are no previous studies on the effect of vitamin D deficiency on the association between shift work and metabolic syndrome. Therefore, this study aimed to investigate the effect of vitamin D deficiency on metabolic syndrome among shift workers.

## Methods

### Ethical considerations

The institutional review board of a university hospital in South Korea approved this study (approval number: KBSMC 2022-03-028). The requirement for informed consent was waived owing to the use of de-identified data.

### Study design and participants

This cross-sectional study was conducted at a large university hospital in South Korea between January 2012 and December 2018. It used data from a cohort of Korean men and women aged ≥18 years who underwent comprehensive health screening examinations at a large university hospital in South Korea (N=496 817). More than 80% of the participants were employees or their dependents who were provided with free annual or biennial health screening examinations guaranteed by the Occupational Safety and Health Act and the National Health Insurance Act. The remaining participants were those who voluntarily paid for their health screening exams. In the case of multiple visits, the first visit was investigated rather than the last visit to prevent an increase in causal interference.

The exclusion criteria were the following: no measurement of vitamin D level; no occupational information; missing data on metabolic syndrome components or covariates; older than 70 years; and history of cancer. In total, 207 756 individuals were included in the analysis (supplementary material, www.sjweh.fi/article/4072, figure S1).

### Measurement of variables

Data on age, sex, date of visit, smoking status, alcohol consumption, education level, marital status, leisure time physical activity level, sleep duration, medication use, and work schedules were obtained using a self-administered questionnaire and reviewed during a face-to-face interview with a doctor. Smoking status was categorized as non-current or current smoker. Alcohol consumption (grams per day) was calculated using the frequency and amount of alcohol consumed per day and categorized into excessive alcohol consumption (≥30 g/day for men and ≥20 g/day for women) and moderate alcohol consumption (<30 g/day for men and <20 g/day for women) (30). Marital status was categorized as not married or married. Education level was categorized into less than college graduate and college graduate or more. Leisure time physical activity level was assessed using the International Physical Activity Questionnaires–Short Form and calculated in terms of metabolic equivalent of task (MET)-minutes per week. One MET is defined as the amount of oxygen consumed while sitting at rest (3.5 mL O_2_ kg^−1^ min^−1^) (31). Sleep duration was investigated using the question “During the past month, how many hours did you sleep at night?”. The use of medications such as antihypertensives, insulin or oral hypoglycemic agents, medication for dyslipidemia, and vitamin D supplements were investigated. Work schedules were investigated using the question “In the past year, during which time of the day have you worked the most?”. The following answers were possible: “I work mostly during the day (06:00–18:00 hours)” and “I work during other hours.” Participants providing the latter response were classified as shift workers, while the others were classified as day workers.

Anthropometric measurements, including height, body weight, waist circumference, and blood pressure were measured by trained nurses with the participants wearing a lightweight hospital gown and no shoes. Body mass index (BMI) was calculated by dividing body weight in kilograms by height in meters squared. Blood parameters were measured using venous blood samples collected from the antecubital vein after ≥10 hours of fasting. The vitamin D level was determined by measuring the 25-hydroxyvitamin D level using a competitive immunoassay, the Elecsys vitamin D total assay, on a Modular E170 immunoanalyzer (Roche, Basel, Switzerland). The limit of detection was 2.0 ng/mL. Serum total cholesterol, high-density lipoprotein cholesterol (HDL-C), low-density lipoprotein cholesterol (LDL-C), and triglyceride levels were measured using an enzymatic colorimetric assay. Serum fasting glucose levels were measured using the hexokinase method on a Cobas Integra 800 apparatus (Roche Diagnostics, Tokyo, Japan). Hemoglobin A1c (HbA1c) level was measured using an immunoturbidimetric assay with a Cobas Integra 800 automatic analyzer (Roche Diagnostics, Tokyo, Japan).

### Definition of metabolic syndrome

Metabolic syndrome was defined as the presence of ≥3 of the following specific criteria (i): waist circumference ≥88 cm and ≥102 cm among women and men, respectively (ii); triglyceride level ≥150 mg/dL or drug treatment for elevated triglyceride levels (iii); HDL-C level <40 mg/dL and <50 mg/dL among men and women, respectively, or drug treatment for low HDL-C levels (iv); systolic blood pressure (SBP) ≥130 mmHg or diastolic blood pressure (DBP) ≥85 mmHg (v); fasting serum glucose level ≥110 mg/dL or drug treatment for elevated glucose levels (32).

### Classification of vitamin D levels

The Institute of Medicine (IOM) committee defined vitamin D levels of <12, 12–20, and ≥20 ng/mL as vitamin D deficient, insufficient, and sufficient status, respectively (33). This study used the IOM classification for vitamin D levels.

### Statistical analysis

For the descriptions of the general characteristics of the study participants, categorical variables were expressed as percentages (%) and were analyzed using the chi-square test. Continuous variables were expressed as mean values with standard deviation (SD) and were analyzed using the t-test. Binary logistic regression was used to determine the association between shift work and metabolic syndrome. Multinomial logistic regression was used to determine the following associations (i): shift work and vitamin D status and (ii) vitamin D status and metabolic syndrome according to work schedules. For each binary or multinomial logistic regression model, the results are expressed as odds ratios (OR) with 95% confidence intervals (CI). Multivariate regression was used to determine the association between continuous vitamin D levels (ng/mL) and metabolic syndrome components. Multivariate regression results were expressed as unstandardized regression coefficients with 95% CI, standardized errors, and standardized regression coefficients. We adjusted for age and sex in Model 1. In addition to these variables, we adjusted for month of visit date, vitamin D supplement use, smoking status, alcohol consumption, leisure time physical activity level, marital status, and education in Model 2. In addition to the variables in Model 2, sleep duration was adjusted for in Model 3. The interaction effect between shift work and vitamin D levels on metabolic syndrome was assessed by calculating the relative excess risk due to interaction (RERI), attributable proportion (AP), and synergy index (S) with excessive odds ratios (EOR), defined as OR – 1, and 95% CI as follows (34):


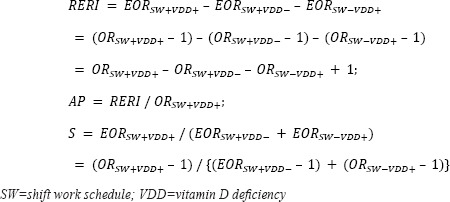


To determine the mediating effect of vitamin D deficiency on the association between shift work and metabolic syndrome, we used the Paramed module developed by Emsley and Liu (35) and conducted mediation analysis using the following logit models:


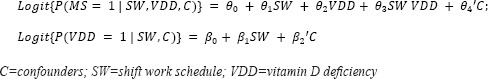


The direct effect was the effect of shift work on metabolic syndrome without the effect of vitamin D, while the indirect effect was mediated by vitamin D. The total effect was estimated as the product of the direct and indirect effects. All statistical analyses were performed using STATA version 16.1 (Stata Corp LP, College Station, TX, USA). Two-tailed P<0.05 were considered statistically significant.

### Patient and public involvement

Patients and/or the public were not involved in the design, conduct, reporting, or dissemination plans of this research.

## Results

### Participant characteristics

The mean age of the participants was 37 (standard deviation 8) years, and the majority (66.3%) of the participants were men. Most of the participants were married (72.3%), college graduate or more (83.1%), and worked mostly during the daytime (87.5%). In total, 5.5% of the participants met the diagnostic criteria of metabolic syndrome, while 27.8% were vitamin D deficient (<12 ng/mL) and 73.6% had vitamin D levels of <20 ng/mL. There were significant differences in age, sex, month of visit date, smoking status, alcohol consumption, education level, marital status, leisure time physical activity level, sleep duration, vitamin D supplement use, BMI, waist circumference, systolic blood pressure, diastolic blood pressure, vitamin D, fasting glucose, HbA1c, total cholesterol, HDL-C, LDL-C, triglyceride levels, and proportion of metabolic syndrome between day workers and shift workers (table 1).

**Table 1:** Baseline characteristics of the participants. Data are presented as number with percentage, N (%) or mean value with standard deviation (SD). [BMI=body mass index; DBP=diastolic blood pressure; HDL-C=high-density lipoprotein cholesterol; LDL-C=low-density lipoprotein-cholesterol; METs=metabolic equivalents of task; SBP=systolic blood pressure]

Characteristics	Total		Work schedule				P-value

			Day work		Shift work		
		
	N (%)	Mean (SD)	N (%)	Mean (SD)	N (%)	Mean (SD)	
Overall	207 756 (100)		181 692 (87.5)		26 064 (12.5)		
Age, years		37 (8)		37 (8)		32 (8)	<0.001
Sex							
Male	137 825 (66.3)		125 025 (68.8)		12 800 (49.1)		<0.001
Female	69 931 (33.7)		56 667 (31.2)		13 264 (50.9)		
Smoking status							
Non-current	160 348 (77.2)		139 074 (76.5)		21 274 (81.6)		<0.001
Current	47 408 (22.8)		42 618 (23.5)		4790 (18.4)		
Excessive alcohol consumption							
No	172 196 (82.9)		151 181 (83.2)		21 015 (80.6)		<0.001
Yes	35 560 (17.1)		30 511 (16.8)		5049 (19.4)		
Education level							
<College	35 108 (16.9)		22 936 (12.6)		12 172 (46.7)		<0.001
≥College	172 648 (83.1)		158 756 (87.4)		13 892 (53.3)		
Marital status							
Not married	57 530 (27.7)		44 851 (24.7)		12 679 (48.7)		<0.001
Married	150 226 (72.3)		136 841 (75.3)		13 385 (51.3)		
Leisure time physical activity level, METs-min/week		1526 (3 161)		1427 (2778)		2216 (5033)	<0.001
Sleep duration at night (hours)							
<5	8728 (4.2)		7244 (4.0)		1484 (5.7)		<0.001
5–6	38 036 (18.3)		34 002 (18.7)		4034 (15.5)		
6–7	83 408 (40.2)		75 511 (41.6)		7897 (30.3)		
≥7	77 584 (37.3)		64 935 (35.7)		12 649 (48.5)		
Vitamin D level (ng/mL)		16.6 (7.1)		16.9 (7.1)		14.7 (6.7)	<0.001
<12	57 661 (27.8)		47 074 (25.9)		10 587 (40.6)		<0.001
12–20	95 306 (45.9)		84 372 (46.4)		10 934 (42.0)		
>20	54 789 (26.4)		50 246 (27.7)		4543 (17.4)		
Metabolic syndrome							
No	196 321 (94.5)		171 408 (94.3)		24 913 (95.6)		<0.001
Yes	11 435 (5.5)		10 284 (5.7)		1151 (4.4)		
Metabolic syndrome components							
BMI (kg/m^2^)	23.6 (3.4)		23.6 (3.4)		23.3 (3.9)		<0.001
Waist circumference (cm)	82 (10)		83 (10)		81 (11)		<0.001
Fasting glucose level (mg/dL)	94 (14)		95 (14)		93 (13)		<0.001
SBP (mmHg)	109 (13)		109 (13)		108 (12)		<0.001
DBP (mmHg)	70 (10)		71 (10)		69 (9)		<0.001
Total cholesterol level (mg/dL)	193 (34)		193 (34)		188 (33)		<0.001
HDL-C level (mg/dL)	58 (15)		58 (15)		61 (16)		<0.001
LDL-C level (mg/dL)	121 (32)		122 (32)		116 (32)		<0.001
Triglyceride level (mg/dL)		114 (80)		116 (81)		99 (71)	<0.001

### Effect of shift work on metabolic syndrome

After adjustments for age and sex, the OR of metabolic syndrome for shift work compared to day work was 1.25 (95% CI 1.17–1.33). Even after adjustment for all confounders, the association remained significant (OR 1.14, 95% CI 1.06–1.22) (table 2).

**Table 2:** Odds ratios (OR) of metabolic syndrome for shift work compared with day work. [Ref=reference]

Work schedule	N case/exposed	OR of metabolic syndrome		

		Model 1 ^a^	Model 2 ^b^	Model 3 ^c^
Day work	10 284/181 692	1 (Ref)	1 (Ref)	1 (Ref)
Shift work	1151/26 064	1.25 (1.17–1.33)	1.14 (1.06–1.22)	1.14 (1.06–1.22)
P-value		<0.001	<0.001	<0.001

a Adjusted for age and sex.

b Adjusted for Model 1 variables plus month of visit date, vitamin D supplement use, smoking status, alcohol consumption, leisure time physical activity level, marital status, and education level.

c Adjusted for Model 2 variables plus sleep duration.

### Effect of shift work on vitamin D levels

When the reference level of vitamin D was ≥20 ng/mL, in the multivariate-fully adjusted model (Model 3), the OR for levels of 12–20 and <12 ng/mL were 1.22 (95% CI 1.17–1.27) and 1.63 (95% CI 1.57–1.70) for shift work compared with day work, respectively (table 3).

**Table 3:** Odds ratio (OR) of vitamin D levels^a^ for shift work compared with day work. [CI=confidence interval; Ref=reference]

	Day work		Shift work		P-value
		
	N	OR (95% CI)	N	OR (95% CI)	
N case/exposed (ng/mL)					
12–20	4773/84 372		518/10 934		
<12	2134/47 074		407/10 587		
Model 1 ^b^(ng/mL)					
12–20		1 (Ref)		1.18 (1.14–1.23)	<0.001
<12		1 (Ref)		1.63 (1.57–1.69)	<0.001
Model 2 ^c^(ng/mL)					
12–20		1 (Ref)		1.21 (1.17–1.26)	<0.001
<12		1 (Ref)		1.62 (1.55–1.69)	<0.001
Model 3 ^d^(ng/mL)					
12–20		1 (Ref)		1.22 (1.17–1.27)	<0.001
<12		1 (Ref)		1.63 (1.57–1.70)	<0.001

a Reference of Vitamin D level: ≥20 ng/mL.

b Adjusted for age and sex.

c Adjusted for Model 1 variables plus month of visit date, vitamin D supplement use, smoking status, alcohol consumption, leisure time physical activity level, marital status, and education level.

d Adjusted for Model 2 variables plus sleep duration.

### Effect of vitamin D levels on metabolic syndrome according to work schedules

In the multivariate-fully adjusted model, there was significant association between vitamin D levels and metabolic syndrome regardless of work schedule. In all workers, the OR of metabolic syndrome were 1.18 (95% CI 1.13–1.24) and 1.35 (95% CI 1.28–1.43) for vitamin D levels of 12–20 and <12 ng/mL compared with ≥20 ng/mL, respectively. In day workers, the OR of metabolic syndrome were 1.17 (95% CI 1.12–1.23) and 1.33 (95% CI 1.25–1.42) for vitamin D levels of 12–20 and <12 ng/mL compared with a level of ≥20 ng/mL, respectively. Among shift workers, the OR of metabolic syndrome were 1.36 (95% CI 1.15–1.61) and 1.51 (95% CI 1.26–1.81) for vitamin D levels of 12–20 and <12 ng/mL compared with a level of ≥20 ng/mL, respectively. All P-values for trends were <0.001 (table 4).

**Table 4:** Odds ratios (OR) of metabolic syndrome for vitamin D levels according to work schedules. [Ref=reference]

Vitamin D	N case/exposed	OR of metabolic syndrome		

		Model 1 ^a^	Model 2 ^b^	Model 3 ^c^
All workers (ng/mL)				
≥20	3603/54 789	1 (Ref)	1 (Ref)	1 (Ref)
12–20	5291/95 306	1.12 (1.07–1.17)	1.19 (1.13–1.24)	1.18 (1.13–1.24)
<12	2541/57 661	1.22 (1.15–1.29)	1.36 (1.28–1.43)	1.35 (1.28–1.43)
P for trend		<0.001	<0.001	<0.001
Day workers (ng/mL)				
≥20	3377/50 246	1 (Ref)	1 (Ref)	1 (Ref)
12–20	4773/84 372	1.11 (1.05–1.16)	1.17 (1.12–1.23)	1.17 (1.12–1.23)
<12	2134/47 074	1.19 (1.12–1.26)	1.34 (1.26–1.42)	1.33 (1.25–1.42)
P for trend		<0.001	<0.001	<0.001
Shift workers (ng/mL)				
≥20	226/10 587	1 (Ref)	1 (Ref)	1 (Ref)
12–20	518/10 934	1.33 (1.12–1.57)	1.36 (1.15–1.61)	1.36 (1.15–1.61)
<12	407/10 587	1.44 (1.20–1.72)	1.50 (1.25–1.81)	1.51 (1.26–1.81)
P for trend		<0.001	<0.001	<0.001

a Adjusted for age and sex.

b Adjusted for Model 1 variables plus month of visit date, vitamin D supplementation, smoking status, alcohol consumption, leisure time physical activity level, marital status, and education level.

c Adjusted for Model 2 variables plus sleep duration.

### Effect of vitamin D levels on metabolic syndrome components

In the multivariate regression analysis, there were inverse associations between vitamin D levels and BMI, waist circumference, fasting glucose, DBP, and triglycerides. The regression coefficients were -0.012 (95% CI -0.014– -0.010), -0.062 (95% CI -0.067– -0.056), -0.040 (95% CI -0.048– -0.032), -0.008 (95% CI -0.014– -0.003), and -0.472 (95% CI -0.519– -0.424), respectively. SBP (mmHg) and HDL (mg/dL) were positively associated with vitamin D levels (ng/mL) and the regression coefficients were 0.014 95% CI (0.007–0.021) and 0.192 (95% CI 0.184–0.201), respectively (supplementary table S1). All the variance inflation factor (VIF) values of variables in the regression model were <10, and the VIF value of vitamin D level was 1.14.

### Interaction between shift work and vitamin D deficiency in metabolic syndrome

There was an additive interaction between shift work and vitamin D deficiency in the development of metabolic syndrome. In the fully adjusted model, the EOR for the combined effect of shift work and vitamin D deficiency was greater by 0.26 (95% CI 0.08–0.44) and 2.09 (95% CI 1.23–3.55) times larger than the sum of the EOR for individual effects. The additional effect due to the interaction accounted for 17% (95% CI 0.07–0.28) of the combined effect (supplementary table S2).

### Mediating effect of vitamin D deficiency on the relationship between shift work and metabolic syndrome

**Figure 1:**
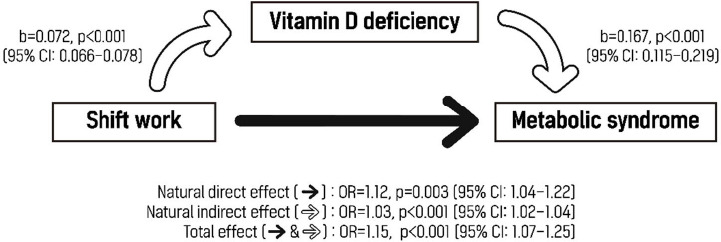
Mediating effect of vitamin D deficiency on the relationship between shift work and metabolic syndrome. Vitamin D deficiency was defined as a vitamin D level <12 ng/mL. The covariates included were age, sex, month of visit date, vitamin D supplement use, smoking status, alcohol consumption, leisure time physical activity level, marital status, education level, and sleep duration. [CI=confidence interval; NDE=natural direct effect; NIE=natural indirect effect; OR=odds ratio; TE=total effect]

There was significant mediating effect of vitamin D deficiency on the association between shift work and metabolic syndrome. The natural direct effect of shift work on metabolic syndrome without the effect of vitamin D deficiency was 1.12 (95% CI 1.04–1.22), while the indirect effect mediated by vitamin D deficiency was 1.03 (95% CI 1.02–1.04). The total effect was 1.15 (95% CI 1.07–1.25), and the indirect effect accounted for 18% of the total effect (figure 1).

## Discussion

In this study, we found that vitamin D partially mediated the relationship between shift work and metabolic syndrome. Shift workers were more likely to be vitamin D deficient than day workers, and vitamin D deficiency increased the risk of metabolic syndrome among shift workers. In addition, there was an additive interaction between shift work and vitamin D deficiency in the development of metabolic syndrome.

The prevalence of vitamin D deficiency varies in different regions of the world since UVB absorption can be affected by geographic latitude, ethnic differences in skin pigmentation, and cultural factors such as clothing (36, 37). Regionally representative population-based studies from the US, Canada, and Europe reported that the prevalence of vitamin D deficiency, defined as vitamin D levels <12 ng/mL (30 nmol/L), was 5.9%, 7.4%, and 13%, respectively, whereas the prevalence of vitamin D deficiency was 27.8% in this study (38–40). This was consistent with previous findings that Asia is one of the regions with the highest prevalence of vitamin D deficiency (36). A recent nationwide study from South Korea reported that 71.8% of the participants had vitamin D levels of <20 ng/mL, which is a similar proportion to our findings (73.6%) (41).

Shift work might be an occupational risk factor for vitamin D deficiency since approximately 90% of vitamin D is synthesized through the exposure of skin to sunlight and shift workers can plausibly be expected to have less exposure to sunlight than day workers (28). Sowah et al (29) evaluated vitamin D levels in different occupations (indoor and outdoor workers, shift workers, lead and smelter workers, and healthcare workers); the results showed that shift workers had the lowest average levels of serum vitamin D (13.5, SD 4.0, ng/mL), and 80% of shift workers had vitamin D levels ≤20 ng/mL, which are similar results to our findings among shift workers (mean levels: 14.7, SD 6.6, ng/mL; 82.6% of shift workers had levels <20 ng/mL). The slight difference might be due to individual factors, such as the actual amount of direct exposure to sunlight, supplementation of vitamin D, and the detailed nature of work such as occupation-specific tasks and types of shift work (42). Although we considered vitamin D supplement use in our study’s analysis, quantitative estimation of sun exposure and detailed work schedules, such as shift work type, were not performed. Further studies are needed to confirm the association between shift work and vitamin D deficiency by considering all of these factors.

Recent meta-analyses support a significant dose-response association between serum vitamin D level and metabolic syndrome. Hajhashemy et al. (43) showed that each 25 nmol/L (10 ng/mL) increase in serum vitamin D levels was significantly associated with 15% decreased odds of metabolic syndrome (OR 0.85; 95% CI 0.80–0.91) in 23 cross-sectional studies in representative populations. Another recent dose–response meta-analysis that was conducted through February 2020 showed that a 25 nmol/L increase in serum vitamin D concentration was associated with a 19% (OR 0.81; 95% CI 0.77–0.85) lower risk of metabolic syndrome in 19 cross-sectional and four cohort studies (44). Our study also showed that vitamin D was associated with metabolic syndrome in a dose–response manner regardless of work schedules. The magnitude of the association was higher among shift workers than among day workers, and it implied an additive interaction between shift work and vitamin D levels in metabolic syndrome.

From a mechanistic perspective, a number of different physical characteristics of metabolic syndrome can be related to vitamin D level status. In our study, vitamin D levels were inversely associated with BMI, waist circumference, fasting glucose, DBP and triglyceride, whereas positively associated with HDL as expected. However, the positive correlation between vitamin D levels and SBP in our study was not consistent with the hypothesis that vitamin D deficiency would increase blood pressure by stimulating RAAS activity (22). Some studies showed a U-shaped relationship between vitamin D levels and SBP rather than a linear inverse correlation. Tomaino et al. showed a U-shaped relationship of vitamin D levels with SBP, while inverse J-shaped relationships with DBP and mean arterial pressure among Peruvian adolescents (45). In an animal study, Mirhosseini et al (46) showed both high and low vitamin D levels can increase SBP through feeding test in normal rats. Further studies are needed to determine the relationship between high vitamin D levels and blood pressure.

Many studies have shown associations of shift work with metabolic syndrome (2–5). Since shift workers were more likely to be vitamin D deficient than day workers and vitamin D deficiency was associated with metabolic syndrome, the mediating effect of vitamin D deficiency in the relationship between shift work and metabolic syndrome can plausibly be expected. However, to the best of our knowledge, no studies have investigated the role of vitamin D in the association between shift work and metabolic syndrome. Our study newly suggests that vitamin D deficiency among shift workers might contribute to the association between shift work and metabolic syndrome. Although some researchers have pointed out that most studies indicating a significant association between shift work and metabolic syndrome lack the consideration of a sleep duration parameter, which can significantly confound the association, our study results were consistent even after adjustment for sleep duration (47). The effect size of shift work for metabolic syndrome was not large in our study. Since metabolic syndrome is characterized as a cluster of cardiovascular risk factors, it can be developed by a number of similarly influential factors rather than very deterministic factor. Therefore, a small effect size of shift work alone is an inevitable result.

In our study, an additive interaction between shift work and vitamin D deficiency in the development of metabolic syndrome was observed as well as a mediating effect of vitamin D deficiency in the association between shift work and metabolic syndrome. Some *in vivo* studies showed that melatonin and vitamin D have synergistic inhibitory action to adipogenesis compared with the effect elicited by each individual molecule (48, 49). Conversely, it can be expected that reduced secretion of melatonin due to shift work and vitamin D deficiency might have a synergistic effect on adipogenesis and thus, metabolic syndrome.

Our study has some limitations. First, its cross-sectional design made it difficult to determine causality. In an effort to minimize reverse causality, we adjusted for potential confounding factors such as lifestyle habits and leisure time physical activity level, and thus reduced the unexpected effects of metabolic syndrome causing decrease in outdoor activity and vitamin D levels. Further longitudinal studies are required to confirm the causality of the relationship between shift work, vitamin D deficiency, and metabolic syndrome. Second, participants in our study were classified into day worker or shift worker through a single questionnaire regarding whether they worked mostly during the day (06:00–18:00 hours) or during other hours in the past year. Therefore, work-related factors, such as shift work type and working environment, were not considered in adequate detail. Further research needs to consider more specific and concrete occupational factors to analyze the effects of shift work. Third, since our study was retrospectively designed using the results of past health examinations, we could not further analyze the effect of correction of vitamin D deficiency through interventions such as supplementation of vitamin D or increasing the duration of sunlight exposure. Fourth, our study was conducted on relatively young and well-educated Korean men and women, and almost all areas of South Korea are located between the latitudes 33°N and 38°N (41). Therefore, despite the large sample size, the generalizability of our findings to other populations needs to be verified.

Nonetheless, we believe that our study has remarkable novelty, as to the best of our knowledge, this is the first large-scale study to evaluate the role of vitamin D in the association between shift work and metabolic syndrome. In addition, our study effectively controlled for potential confounding factors by adjusting for multiple demographic and clinical factors.

### Concluding remarks

This cross-sectional study verified the mediating role of vitamin D in the association between shift work and metabolic syndrome, showing a synergistic interaction between shift work and vitamin D deficiency in metabolic syndrome. Further prospective studies are required to confirm the effect of vitamin D deficiency on metabolic syndrome among shift workers.

### Financial material support

The authors received no specific funding for this study.

### Conflicts of interest

The authors declare no conflicts of interest.

## Supplementary material

Supplementary material

## References

[1] Saklayen MG (2018). The global epidemic of the metabolic syndrome. Curr Hypertens Rep.

[2] Wang Y, Yu L, Gao Y, Jiang L, Yuan L, Wang P (2021). Association between shift work or long working hours with metabolic syndrome:a systematic review and dose-response meta-analysis of observational studies. Chronobiol Int.

[3] Yang X, Di W, Zeng Y, Liu D, Han M, Qie R (2021). Association between shift work and risk of metabolic syndrome:A systematic review and meta-analysis. Nutr Metab Cardiovasc Dis.

[4] Khosravipour M, Khanlari P, Khazaie S, Khosravipour H, Khazaie H (2021). A systematic review and meta-analysis of the association between shift work and metabolic syndrome:the roles of sleep, gender, and type of shift work. Sleep Med Rev.

[5] Wang F, Zhang L, Zhang Y, Zhang B, He Y, Xie S (2014). Meta-analysis on night shift work and risk of metabolic syndrome. Obes Rev.

[6] Garaulet M, Madrid JA (2009). Chronobiology, genetics and metabolic syndrome. Curr Opin Lipidol.

[7] Peixoto AJ, White WB (2007). Circadian blood pressure:clinical implications based on the pathophysiology of its variability. Kidney Int.

[8] Corbalán-Tutau D, Madrid JA, Nicolás F, Garaulet M (2014). Daily profile in two circadian markers “melatonin and cortisol”and associations with metabolic syndrome components. Physiol Behav.

[9] Vinogradova I, Anisimov V (2013). Melatonin prevents the development of the metabolic syndrome in male rats exposed to different light/dark regimens. Biogerontology.

[10] Kitagawa A, Ohta Y, Ohashi K (2012). Melatonin improves metabolic syndrome induced by high fructose intake in rats. J Pineal Res.

[11] Wei T, Li C, Heng Y, Gao X, Zhang G, Wang H (2020). Association between night-shift work and level of melatonin:systematic review and meta-analysis. Sleep Med.

[12] Fonken LK, Workman JL, Walton JC, Weil ZM, Morris JS, Haim A (2010). Light at night increases body mass by shifting the time of food intake. Proc Natl Acad Sci USA.

[13] Holmbäck U, Forslund A, Lowden A, Forslund J, Akerstedt T, Lennernäs M (2003). Endocrine responses to nocturnal eating--possible implications for night work. Eur J Nutr.

[14] Xie J, Li Y, Zhang Y, Vgontzas AN, Basta M, Chen B (2021). Sleep duration and metabolic syndrome:an updated systematic review and meta-analysis. Sleep Med Rev.

[15] Walsh JS, Bowles S, Evans AL (2017). Vitamin D in obesity. Curr Opin Endocrinol Diabetes Obes.

[16] Wortsman J, Matsuoka LY, Chen TC, Lu Z, Holick MF (2000). Decreased bioavailability of vitamin D in obesity. Am J Clin Nutr.

[17] Targher G, Bertolini L, Scala L, Cigolini M, Zenari L, Falezza G (2007). Associations between serum 25-hydroxyvitamin D3 concentrations and liver histology in patients with non-alcoholic fatty liver disease. Nutr Metab Cardiovasc Dis.

[18] Wamberg L, Christiansen T, Paulsen SK, Fisker S, Rask P, Rejnmark L (2013). Expression of vitamin D-metabolizing enzymes in human adipose tissue -- the effect of obesity and diet-induced weight loss. Int J Obes.

[19] Vranić L, Mikolašević I, Milić S (2019). Vitamin D deficiency:consequence or cause of obesity?Medicina (Kaunas).

[20] Wood RJ (2008). Vitamin D and adipogenesis:new molecular insights. Nutr Rev.

[21] Park JE, Pichiah PB, Cha YS (2018). Vitamin D and metabolic diseases:growing roles of vitamin D. J Obes Metab Syndr.

[22] Li YC, Qiao G, Uskokovic M, Xiang W, Zheng W, Kong J (2004). Vitamin D:a negative endocrine regulator of the renin-angiotensin system and blood pressure. J Steroid Biochem Mol Biol.

[23] Martin T, Campbell RK (2011). Vitamin D and diabetes. Diabetes Spectr.

[24] Gagnon C, Daly RM, Carpentier A, Lu ZX, Shore-Lorenti C, Sikaris K (2014). Effects of combined calcium and vitamin D supplementation on insulin secretion, insulin sensitivity and β-cell function in multi-ethnic vitamin D-deficient adults at risk for type 2 diabetes:a pilot randomized, placebo-controlled trial. PLoS One.

[25] Gao Y, Wu X, Fu Q, Li Y, Yang T, Tang W (2015). The relationship between serum 25-hydroxy vitamin D and insulin sensitivity and β-cell function in newly diagnosed type 2 diabetes. J Diabetes Res.

[26] Park S, Kim DS, Kang S (2016). Vitamin D deficiency impairs glucose-stimulated insulin secretion and increases insulin resistance by reducing PPAR-γexpression in nonobese Type 2 diabetic rats. J Nutr Biochem.

[27] Szymczak-Pajor I, Śliwińska A (2019). Analysis of association between vitamin D deficiency and insulin resistance. Nutrients.

[28] Bogh MK (2012). Vitamin D production after UVB:aspects of UV-related and personal factors. Scand J Clin Lab Invest Suppl.

[29] Sowah D, Fan X, Dennett L, Hagtvedt R, Straube S (2017). Vitamin D levels and deficiency with different occupations:a systematic review. BMC Public Health.

[30] Okechukwu CA (2015). Long working hours are linked to risky alcohol consumption. BMJ.

[31] Craig CL, Marshall AL, Sjöström M, Bauman AE, Booth ML, Ainsworth BE (2003). International physical activity questionnaire:12-country reliability and validity. Med Sci Sports Exerc.

[32] Expert Panel on Detection, Evaluation, and Treatment of High Blood Cholesterol in Adults (2001). Executive Summary of The Third Report of The National Cholesterol Education Program (NCEP) Expert Panel on Detection, Evaluation, And Treatment of High Blood Cholesterol In Adults (Adult Treatment Panel III). JAMA.

[33] Ross AC, Manson JE, Abrams SA, Aloia JF, Brannon PM, Clinton SK (2011). The 2011 report on dietary reference intakes for calcium and vitamin D from the Institute of Medicine:what clinicians need to know. J Clin Endocrinol Metab.

[34] Knol MJ, VanderWeele TJ, Groenwold RH, Klungel OH, Rovers MM, Grobbee DE (2011). Estimating measures of interaction on an additive scale for preventive exposures. Eur J Epidemiol.

[35] Emsley R, Liu H (2013). PARAMED:Stata module to perform causal mediation analysis using parametric regression models [Internet].

[36] Roth DE, Abrams SA, Aloia J, Bergeron G, Bourassa MW, Brown KH (2018). Global prevalence and disease burden of vitamin D deficiency:a roadmap for action in low- and middle-income countries. Ann N Y Acad Sci.

[37] Cashman KD (2020). Vitamin D deficiency:defining, prevalence, causes, and strategies of addressing. Calcif Tissue Int.

[38] Schleicher RL, Sternberg MR, Looker AC, Yetley EA, Lacher DA, Sempos CT (2016). National estimates of serum total 25-hydroxyvitamin D and metabolite concentrations measured by liquid chromatography-tandem mass spectrometry in the US Population during 2007 2010. J Nutr.

[39] Sarafin K, Durazo-Arvizu R, Tian L, Phinney KW, Tai S, Camara JE (2015). Standardizing 25-hydroxyvitamin D values from the Canadian Health Measures Survey. Am J Clin Nutr.

[40] Cashman KD, Dowling KG, Škrabáková Z, Gonzalez-Gross M, Valtueña J, De Henauw S (2016). Vitamin D deficiency in Europe:pandemic?Am J Clin Nutr.

[41] Park JH, Hong IY, Chung JW, Choi HS (2018). Vitamin D status in South Korean population:seven-year trend from the KNHANES. Medicine (Baltimore).

[42] Coppeta L, Papa F, Magrini A (2018). Are shiftwork and indoor work related to D3 vitamin deficiency?A systematic review of current evidences. J Environ Public Health.

[43] Hajhashemy Z, Shahdadian F, Moslemi E, Mirenayat FS, Saneei P (2021). Serum vitamin D levels in relation to metabolic syndrome:A systematic review and dose-response meta-analysis of epidemiologic studies. Obes Rev.

[44] Lee K, Kim J (2021). Serum vitamin D status and metabolic syndrome:a systematic review and dose-response meta-analysis. Nutr Res Pract.

[45] Tomaino K, Romero KM, Robinson CL, Baumann LM, Hansel NN, Pollard SL, PURA study investigators (2015). Association Between Serum 25-Hydroxy Vitamin D Levels and Blood Pressure Among Adolescents in Two Resource-Limited Settings in Peru. Am J Hypertens.

[46] Mirhosseini NZ, Knaus SJ, Bohaychuk K, Singh J, Vatanparast HA, Weber LP (2016). Both high and low plasma levels of 25-hydroxy vitamin D increase blood pressure in a normal rat model. Br J Nutr.

[47] Canuto R, Garcez AS, Olinto MT (2013). Metabolic syndrome and shift work:a systematic review. Sleep Med Rev.

[48] Basoli V, Santaniello S, Cruciani S, Ginesu GC, Cossu ML, Delitala AP (2017). Melatonin and vitamin D interfere with the adipogenic fate of adipose-derived stem cells. Int J Mol Sci.

[49] Santaniello S, Cruciani S, Basoli V, Balzano F, Bellu E, Garroni G (2018). Melatonin and vitamin D orchestrate adipose derived stem cell fate by modulating epigenetic regulatory genes. Int J Med Sci.

