# Supplementary material for: Effect of vitamin D deficiency on metabolic syndrome among Korean shift workers
Source: Scand J Work Environ Health. 2023 Feb 27;49(2):126–35. doi: 10.5271/sjweh.4072 (PMC10577018; doi:10.5271/sjweh.4072)
Supplement: Supplementary material [file SJWEH-49-126-S001.pdf]

Effect of vitamin D deficiency on metabolic syndrome among Korean shift workers[1]

by Eunchan Mun, MD, Yesung Lee, MD, Woncheol Lee, MD, Soyoung Park, MD [2]

1. SUPPLEMENTARY MATERIAL
2. Correspondence to: Department of Occupational and Environmental Medicine, Kangbuk Samsung Hospital, Sungkyunkwan University, School of Medicine, 29, Saemunan-ro, Jongno-gu, Seoul 03181, Republic of Korea. [E-mail: syoem.park@gmail.com]

Supplemental Table S1. Multiple regression analysis results between continuous vitamin D levels (ng/mL) and metabolic syndrome components

Metabolic syndrome components	Statistics for multiple regression analysis on vitamin D levels (ng/mL) affecting the change in metabolic syndrome components			
	Coefficient (95% CI)	SE	*P*-value	Beta
BMI (kg/m^2)	-0.012 (-0.014 – -0.010)	0.001	<0.001	-0.025
Waist circumference (cm)	-0.062 (-0.067 – -0.056)	0.003	<0.001	-0.044
Fasting glucose (mg/dL)	-0.040 (-0.048 – -0.032)	0.004	<0.001	-0.020
SBP (mmHg)	0.014 (0.007 – 0.021)	0.004	<0.001	0.008
DBP (mmHg)	-0.008 (-0.014 – -0.003)	0.003	0.003	-0.006
HDL (mg/dL)	0.192 (0.184 – 0.201)	0.004	<0.001	0.088
Triglyceride (mg/dL)	-0.472 (-0.519 – -0.424)	0.024	<0.001	-0.042

The regression coefficients were adjusted for age, sex, month of visit date, vitamin D supplement use, smoking status, alcohol consumption, leisure time physical activity level, marital status, education level, sleep duration, and medication for diabetes, hypertension, and dyslipidemia
Beta indicated a standardized regression coefficient
Abbreviation: CI, confidence interval; SE, standardized error, BMI, body mass index; SBP, systolic blood pressure; DBP, diastolic blood pressure; HDL, high density lipid

Supplemental Table S2. Interaction between shift work and vitamin D deficiency in the development of metabolic syndrome

Variables	Interaction index		
	RERI (95% CI)	AP (95% CI)	S (95% CI)
Shift work and vitamin D deficiency (<12 ng/ml)	0.26 (0.08–0.44)	0.17 (0.07–0.28)	2.09 (1.23–3.55)
P-value	0.005	0.002	0.006

The odds ratios were adjusted for age, sex, month of visit date, vitamin D supplement use, smoking status, alcohol consumption, leisure time physical activity level, marital status, education level, and sleep duration

Abbreviation: AP, attributable proportion; RERI, relative excess risks due to interaction; S, synergy index

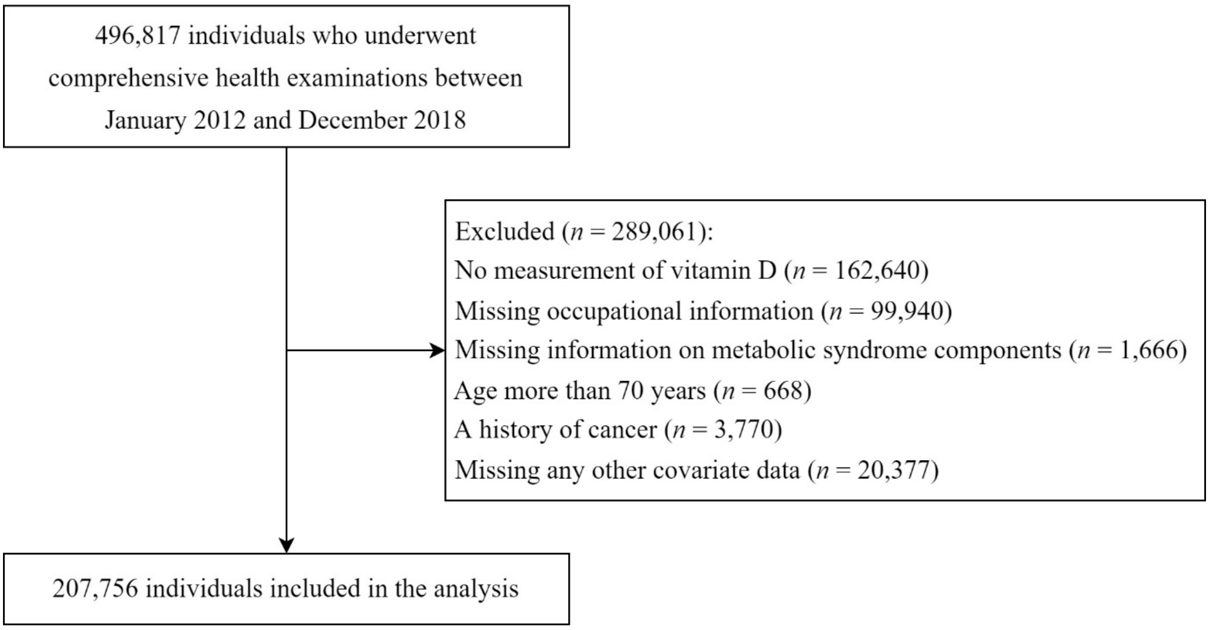

Supplemental Figure S1. Participant selection flowchart
